# Compulsory Vaccination

**Published:** 1911-08

**Authors:** 


### Compulsory Vaccination

The public hearings in the Common Council Chamber on Vaccination have led to the organisation of an anti-vaccination Society. It would seem that the terrible warning of history ought to suffice to convince any thinking person of the value of vaccination. Before the advent of vaccination, smallpox prevailed as commonly as scarlet fever and, like any prevalent semelincident disease to which natural immunity is an exceptional occurrence, mainly as a disease of childhood. Its mortality was great and the disfigurement among survivors was so common that contemporaries spoke of the "waffle-face" as conspicuous by its absence.

We forget these lessons in these days when a physician of long years of extensive practice may admit without shame that he has never seen a case and when the ignorant and negligent are protected by the precautions so long and so generally in force. There seems to be developing, a class of the community that forms opinions without personal knowledge and contrary to the experience of those competent to judge. Such persons must look into a gun to see if it is loaded and put their own finger on the invisible teeth of the buzz saw. This is an expensive way of gaining wisdom. It is claimed that to ascribe the marked diminution of smallpox to vaccination is merely an illustration of the "post hoc, propter hoc" fallacy. Unfortunately, "post hoc, propter hoc," has come to suggest a fallacy immediately whereas the general sequence of one event upon another, nine times out of ten, really signifies a genuine causal relation and not a fallacy.

The diminution of smallpox is ascribed to hygienic factors of a general nature, yet such factors have produced very slight alterations in the incidence of measles and scarlet fever, which are closely analogous clinically and, in all probability, pathogenically.

Aside from the humanitarian sentiment which makes each man and, especially each trained man, his brother's keeper, it would be a valuable experiment to allow the development of a non-immune class. We should then know just how much truth there is in the prevalent conception of anti-variolar immunisation, how far smallpox has been controlled by vaccination and how far by general sanitary measures, how far vaccination without quarantine, can be depended on. Obviously, until opportunities for contracting smallpox have gradually developed, the anti-vaccinationists will be able to say "I told you so."

The personnel of the anti-vaccinationists indicates a strong Christian Science influence and it must be conceded that, from the standpoint of this cult, vaccination, like everything else of a material practical nature, is "error."

We are somewhat at a loss to account for the names of prominent homoeopathic physicians among the list of officers of the Anti-vaccination Society. Vaccination seems to illustrate most admirably the principle of "similia similibus curantur." The virus is now commonly regarded as an attenuated bearer of the variola microörganism and, indeed, certain strains of vaccine are directly traceable to human smallpox. The successive cultures, taking a minute particle of virus from one animal, inoculating another, taking a minute particle from the latter and so on, in a long series, corresponds with almost mathematic accuracy to the preparation of high potentials of an ordinary drug, vaccinia being a miniature variola corresponds to the essentially homoeopathic method of "proving" a therapeutic agent, and the dosage is comparatively minute. Smallpox comes well within the "itch-mite" theory of causation of disease, and for this theory, we believe that it is unfair to ridicule Hahnemann. On the contrary, he should receive credit for having early presented the essential conception of the germ theory of disease, in crude form it is true, but with no more crudity than was inevitable from the comparatively undeveloped state of biologic science in his time. Nor should homoeopathists oppose vaccination on esthetic grounds for, within a very few years, other forms of pus and excrementitious matter from human sources, have been listed by their drug houses.

We cannot but feel that the recent sentiment against vaccination is largely due to the general dissemination of scientific knowledge in so dilute a degree as to lead to arrogance. It requires a good deal of study to make a man humble and receptive of authority. Perhaps, too, Americans are beginning to realize that there is actually less personal liberty in the United States than in any other civilized country of the world between Russia and China and they are taking an unwise mode of protesting against official interference in the details of their private lives.

